# Evaluation of the Effects of Developmental Trauma on Neurotransmitter Systems Using Functional Molecular Imaging

**DOI:** 10.3390/ijms22052522

**Published:** 2021-03-03

**Authors:** Namhun Lee, Se-Jong Oh, Jang-Woo Park, Kyung-Rok Nam, Kyung-Jun Kang, Kyo-Chul Lee, Yong-Jin Lee, June-Seek Choi, Jeong-Ho Seok, Jae-Yong Choi

**Affiliations:** 1 Division of Applied RI, Korea Institute of Radiological and Medical Sciences, Seoul 01812, Korea; nh131@kirams.re.kr (N.L.); osj5353@kirams.re.kr (S.-J.O.); krnam@kirams.re.kr (K.-R.N.); kangkj1@kirams.re.kr (K.-J.K.); kyochul@kirams.re.kr (K.-C.L.); yjlee@kirams.re.kr (Y.-J.L.); 2 Korea Drug Development Platform Using Radio-Isotope, Korea Institute of Radiological and Medical Sciences, Seoul 01812, Korea; jangwoo@kirams.re.kr; 3 Department of Psychology, Korea University, Seoul 02841, Korea; j-schoi@korea.ac.kr; 4 Department of Psychiatry, Yonsei University College of Medicine, Seoul 06273, Korea; 5 Radiological and Medico-Oncological Sciences, University of Science and Technology (UST), Seoul 01812, Korea

**Keywords:** trauma, early life stress, neurotransmission, positron emission tomography

## Abstract

Early life stress (ELS) is strongly associated with psychiatric disorders such as anxiety, depression, and schizophrenia in adulthood. To date, biological, behavioral, and structural aspects of ELS have been studied extensively, but their functional effects remain unclear. Here, we examined NeuroPET studies of dopaminergic, glutamatergic, and serotonergic systems in ELS animal models. Maternal separation and restraint stress were used to generate single or complex developmental trauma. Body weights of animals exposed to single trauma were similar to those of control animals; however, animals exposed to complex trauma exhibited loss of body weight when compared to controls. In behavioral tests, the complex developmental trauma group exhibited a decrease in time spent in the open arm of the elevated plus-maze and an increase in immobility time in the forced swim test when compared to control animals. In NeuroPET studies, the complex trauma group displayed a reduction in brain uptake values when compared to single trauma and control groups. Of neurotransmitter systems analyzed, the rate of decrease in brain uptake was the highest in the serotonergic group. Collectively, our results indicate that developmental trauma events induce behavioral deficits, including anxiety- and depressive-like phenotypes and dysfunction in neurotransmitter systems.

## 1. Introduction

Trauma is an emotional response to stressful events that overwhelm an individual’s coping ability. Trauma can cause helplessness, loss of control, and confusion in later life. Early-life stress (ELS), also known as childhood trauma, may constitute physical, emotional, and sexual abuse; these events exert strong physical and psychological sequelae that persist into adulthood [1]. ELS may increase the risk of adult psychiatric disorders such as anxiety, mood disorders, personality disorders, and posttraumatic stress disorder [2,3,4,5]. Stress during developmental periods leads to a decrease in neurogenesis, dysfunction of neurotransmitter systems, and activation of the hypothalamic–pituitary–adrenal (HPA) axis, which generates changes in neuroplasticity and behavioral deficits [6]. According to the WHO World Mental Health survey in 2010, childhood adversity was associated with approximately 30% of psychiatric disorders in adulthood across 21 countries [7]. This ratio is expected to increase in the future with growing social issues. Thus, understanding the psychopathology of developmental trauma is critical.

Previous studies have examined the effects of ELS on biological, behavioral, and structural domains in adulthood. ELS exerts adverse effects on the release of neurotrophic factors, including brain-derived neurotrophic factor, nerve growth factor, and neurotrophin [8,9]. It also causes brain atrophy in the amygdala and hippocampus and behavioral deficits, including anxiety- and depressive-like phenotypes [10,11,12,13]. However, relatively little is known about the functional changes in neurotransmitter systems underlying these effects. Therefore, a comprehensive evaluation of neurotransmitter systems is necessary to deepen our understanding of developmental trauma.

Positron emission tomography (PET) is a molecular imaging technique that enables the detection of cellular changes in vivo. PET generates a non-invasive assessment of biochemical changes in the body using radiotracers that bind to specific biomarkers, such as receptors, transporters, or pathological deposits in the brain [14,15]. To address the functional changes following ELS in the adult brain, we examined NeuroPET in trauma-exposed and control animals. The aim of this study was to determine the characteristics of complex developmental trauma relative to those of single trauma or control events. It is well established that ELS affects neural responses to emotional and reward processing [16,17]. Therefore, we focused on dopaminergic, glutamatergic, and serotonergic systems, which are closely associated with reward, anxiety, and depression.

## 2. Results

### 2.1. Body Weight and Corticosterone Levels

Animals were weighed, and blood was collected on postnatal day (PND) 55 before molecular imaging studies. No significant differences were observed in body weights of animals in the single trauma groups exposed to either maternal stress (MS) or restraint stress (RS) compared to the body weights of control animals (body weights of control: 402.9 ± 36.9 g vs. MS 388.7 ± 19.4 g, *p* > 0.999; control vs. RS: 403.7 ± 39.0 g, *p* > 0.999). However, animals in the complex trauma (MRS) group lost 30% of their body weight when compared to control animals (control vs. MRS: 281.1 ± 48.0, *p* = 0.0079). Serum corticosterone levels exhibited a decrease in all trauma groups compared to those of the control group; these differences were not statistically significant (Table 1).

### 2.2. Behavioral Tests

#### 2.2.1. Elevated Plus Maze

**Figure 1:**
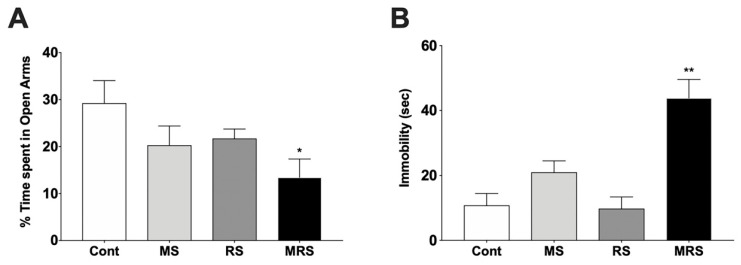
Comparison of the % time spent in the open arms in the elevated plus-maze (**A**) and immobility time in the forced swim test (**B**). Data are presented as means ± SEM (*n* = 5). Statistical significance was defined as a *p*-value less than 0.05 (* *p* < 0.05, ** *p* < 0.01).

The percentage of time spent in the open arms of the elevated plus-maze was lower for all experimental groups than for the control group. Time spent in the open arms by the single trauma MS and RS groups was less than that in the control group, respectively (% time spent in open arms for control: 29.25 ± 10.69 vs. MS 20.28 ± 9.18, *p* = 0.0952; control vs. RS: 21.73 ± 4.47, *p* = 0.1508). Time spent in the open arms by the complex trauma group was less than that of the control group; this difference was statistically significant (control vs. MRS: 13.46 ± 8.81, *p* = 0.0317, Figure 1A).

#### 2.2.2. Forced Swim Test

The immobility time of the MS group in the forced swim test was higher than that of controls without statistical significance. Immobility time of the RS and control groups were similar (immobility time for controls: 10.81 ± 8.17 vs. MS 20.98 ± 7.04, *p* = 0.1905; control vs. RS: 9.79 ± 8.06, *p* = 0.8413). The complex trauma group exhibited an increase in immobility time compared to that of controls; this difference was statistically significant (control vs. MRS: 43.86 ± 12.77, *p* = 0.0079, Figure 1B).

### 2.3. NeuroPET

#### 2.3.1. Dopaminergic System

To evaluate the dopaminergic system, we measured the density of the dopamine D2 receptor (D2R). As D2R is highly expressed in the striatum, we selected this area as the volume of interest (VOI). For the single trauma MS and RS groups, radioactivity in the striatum was higher than that of the control group, respectively (SUV for control: 2.94 ± 0.27 vs. MS 3.10 ± 0.90, *p* = 0.7133; control vs. for RS: 3.37 ± 0.35, *p* = 0.0613). In contrast, uptake was lower in the complex trauma group compared to that in the control group (SUV for control vs. MRS: 2.29 ± 0.32, *p* = 0.0084).

#### 2.3.2. Glutamatergic System

To evaluate the glutamatergic system, we measured the density of the metabotropic glutamate receptor 5 (mGluR5). mGluR5 is expressed in the cerebral cortex, striatum, and hippocampus. The overall radioactivity uptake pattern in cortical and sub-cortical areas decreased in the following order: control > MS > RS > MRS. The complex trauma group exhibited significantly decreased uptake when compared to the control group. In the cortex, single and complex stress groups exhibited lower radioactivity compared to that of the control group (SUV for control: 2.64 ± 0.25 vs. for MS 2.09 ± 0.37, *p* = 0.0249; control vs. for RS 1.94 ± 0.13, *p* = 0.0005; control vs. for MRS 1.91 ± 0.20, *p* = 0.0009). In the striatum, stress exposure groups exhibited lower uptakes compared to that of the control group (SUV for control: 4.64 ± 0.58 vs. for MS 4.08 ± 0.77, *p* = 0.2301; control vs. for RS 3.82 ± 0.28, *p* = 0.0216; MRS 3.66 ± 0.22, *p* = 0.0069). Radioactivity in the hippocampus was lower in stress exposure groups compared to that in the control group (SUV for control: 3.98 ± 0.62 vs. MS 3.12 ± 0.68, *p* = 0.0700; control vs. RS 2.95 ± 0.25, *p* = 0.0088; control vs. MRS 3.11 ± 0.25, *p* = 0.0196).

#### 2.3.3. Serotoninergic System

**Figure 2:**
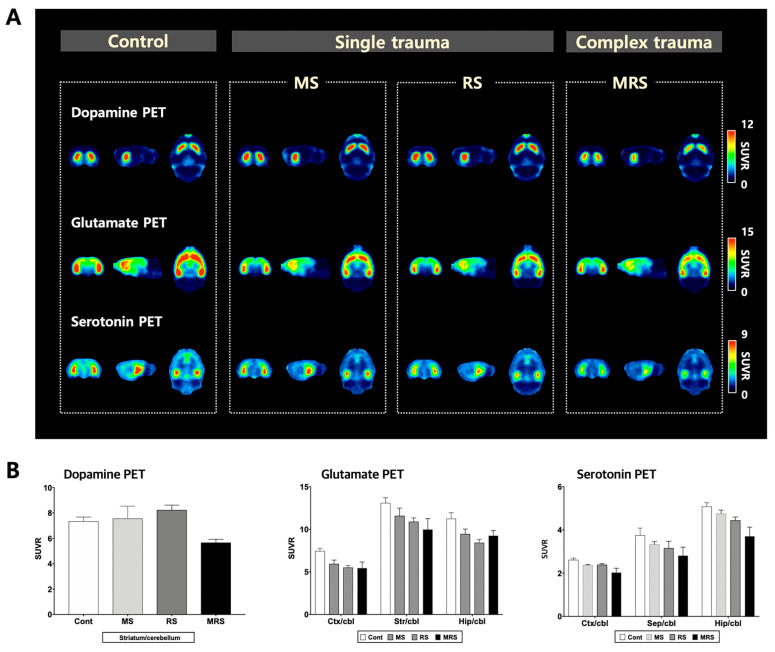
Comparison of neuroreceptor positron emission tomography (PET) images (**A**). The top, middle, and bottom rows indicate dopamine, glutamate, and serotonin PET, respectively. The mean PET images from 40–60 min were obtained from dynamic PET. Each neuroreceptor PET is an average PET image of five individuals. (**B**) Quantification of neuroreceptor PET radioactivity. Regions of interest in PET are included in the striatum (Str), cortex (Ctx), hippocampus (Hip), and cerebellum (cbl). Here, the cerebellum was used as a reference region in PET. Data are presented as means ± SEM (*n* = 5).

To evaluate the serotonergic system, we measured the density of the serotonin 1A receptor (5-HT_1A_). 5-HT_1A_ receptor PET images of the regions of interest, including the cortex, hippocampus, and septum, displayed a decreasing pattern in the order of control, MS, RS, and MRS groups (Figure 2). In the cortex, uptake in the stress exposure groups was lower than that of the control group (SUV for control: 1.35 ± 0.09 vs. MS 1.22 ± 0.04, *p* = 0.0184; control vs. RS 1.22 ± 0.03, *p* = 0.0155; control vs. MRS 1.04 ± 0.10, *p* = 0.0009). In the septum, the stress exposure groups exhibited lower uptake compared to that of the control group (SUV for control: 1.95 ± 0.39 vs. MS 1.71 ± 0.16, *p* = 0.2387; control vs. RS 1.63 ± 0.36, *p* = 0.2145; control vs. MRS 1.44 ± 0.18, *p* = 0.0290). Hippocampal uptake was lower than that in the other brain regions within the trauma groups. Uptake in the stress exposure groups was lower than that in the control group (SUV for control: 2.63 ± 0.20 vs. MS 2.44 ± 0.19, *p* = 0.1621; control vs. RS 2.29 ± 0.16, *p* = 0.0179; control vs. MRS 1.90 ± 0.22, *p* = 0.0006; Figure 2A,B, supplementary information Figure S1 and Table S1).

## 3. Discussion

Early life stress (ELS), which exceeds an individual’s coping ability during infancy and childhood, has a negative effect on mental health in adulthood. Complex developmental trauma, a specific form of ELS, is defined by multiple, chronic, and repetitive traumatic experiences during the developmental period. Although neurobiological and behavioral studies provide convincing evidence for the effects of ELS, underlying changes in brain function have not been fully elucidated. In this study, we aimed to elucidate the changes in neurotransmitter systems in the brain induced by ELS. In particular, we investigated the characteristics of complex developmental trauma when compared to a single trauma or control events using non-invasive molecular imaging. NeuroPET findings indicated that the complex trauma group displayed significantly reduced brain uptake values when compared to the single trauma or control groups. Among these neurotransmitters, the rate of decrease in brain uptake was the highest in the serotonergic system; therefore, this system appears to be the most vulnerable to stress. To the best of our knowledge, this is the first study to show that complex developmental trauma leads to functional neurotransmission changes in vivo.

In this study, MS and RS were used to produce animal models of developmental trauma. In single trauma groups subjected to MS or RS, no significant weight loss differences were observed when compared to the control group. However, the complex developmental trauma group exhibited a significant loss of body weight when compared to the control group. Weight loss may be attributed to a decline in pleasure-seeking from eating, and a decrease in weight in the complex stress group may imply anhedonia. Measurement of serum corticosterone levels after 30 days of stress revealed that corticosterone levels in the trauma-exposed groups were lower than those in the control group. This implies that severe stress during developmental periods may reduce HPA axis activity in adulthood. Nienke et al. reported that corticosterone levels in the MS group increased until juvenile age and subsequently decreased in adulthood [18]. Behaviorally, the complex trauma group exhibited significantly greater anxiety-like and depressive-like phenotypes, in accordance with previous research [19,20,21].

From a neuroimaging perspective, most studies have focused on structural changes based on MRI, with reduced hippocampal volume emerging as a common finding [22,23]. Disease pathogenesis is underpinned by a specific genetic background and biochemical changes, which in turn induce functional and anatomical changes, subsequently manifesting as clinical symptoms. Therefore, examining functional changes in the brain is important because symptoms may occur prior to their manifestation. PET is a commonly used imaging modality because it can identify changes at the cellular level due to its high sensitivity (pico-molar sensitivity). To date, functional changes following the effects of ELS on mental health in adulthood are not well understood. Previous PET studies have predominantly focused on glucose metabolism, serotoninergic and dopaminergic systems. Simona et al. reported that [^18^F]FPWAY PET in monkeys subjected to MS early in life exhibited lower serotonin PET uptake compared to that in the control group [24]. Masanori et al. reported decreased striatal serotonergic PET uptake in the non-maternal care group with [^11^C]DASB [25]. In addition, James et al. conducted a [^11^C]P943 PET for 5-HT1B in early trauma patients and observed that these patients had lower binding in the caudate, amygdala, and anterior cingulate cortex when compared to the control group [26]. Jens et al. reported that the low maternal care group exhibited significantly reduced dopaminergic PET uptake compared to that in the high parental care group [27]. In terms of glucose metabolism, Lisa et al. reported that maternally separated monkeys exhibited lower glucose activity in the hippocampus compared to that in the control group [28]. In the dopamine PET, we observed that brain uptake in the single stressed group (MS or RS) was relatively higher than that in the control group, while brain uptake in the complex trauma group was lower than that in the control group. The single stress used in the present study can lead to a temporary rise in available D2R receptors. Interestingly, this result was in accordance with previous neuroimaging studies in maternally separated monkeys [29]. However, when a complex developmental stress environment is sustained, it can induce neuronal degeneration and, eventually, downregulation of D2R receptors. To address this issue, further studies related to the stress severity and duration are required. Nevertheless, previous PET studies are limited in that only a single biomarker was evaluated in a single trauma model. In contrast, the present study evaluated the changes in mood-related neurotransmitter systems in both single and complex trauma models, which provides a novel perspective.

Repetitive and chronic stress in early life induces hyperactivation of the HPA axis, resulting in the release of corticosterone. Sustained high levels of corticosterone cause a reduction in neurogenesis and neuronal cell death [30,31]. In addition, chronic stress reduces the expression of brain-derived neurotrophic factors, which plays a pivotal role in the promotion of dendritic branching [32]. Given that synaptogenesis is completed within PND 20 [33], ELS may interfere with this process. Zhu et al. reported that chronic mild stress-induced spine loss in dentate gyrus granule cells [34]. These findings suggest that complex developmental trauma may lead to the weakening of specific neurotransmitter systems, which resulted in reduced brain uptake of radiotracers in this study.

This study has several limitations. First, the decrease in brain uptake indicates a change in available receptor density and does not reflect a change in the concentration of neurotransmitters present in synapses. Second, the relationship between anatomical and functional changes in specific brain regions related to mood remains unclear. Finally, the current study used a small sample size and did not evaluate changes in HPA axis-related hormones or perform histological analysis. Therefore, additional studies are required to clarify these issues.

In conclusion, developmental trauma leads to dysfunction in behavior and neurotransmitter systems. In particular, the complex developmental trauma group exhibited increased anxiety- and depressive-like behaviors. From a neurotransmitter perspective, the complex trauma group displayed significantly reduced brain uptake values of NeuroPET tracers when compared with the single trauma or control groups. These results expand our understanding of the psychopathology of developmental trauma.

## 4. Materials and Methods

### 4.1. Animals

Sprague-Dawley rats (*n* = 3) at 2 weeks of gestation were obtained from DooYeol Biotech Co. (Seoul, Korea), and the females were individually housed. The rats were checked daily for birth before 9:00 AM. If pups were identified, they were considered to have been born the day before. The birth day was assigned as postnatal day (PND) 0, and pups were left undisturbed on this day. On PND 1, female pups were sacrificed, and only male pups were cross-fostered to exclude the potential effects of estrogen and different anxiety levels on dams. On PND 2, male pups were randomly assigned to four groups: maternal separation (MS, *n* = 7), restraint stress (RS, *n* = 7), complex trauma (MRS, *n* = 7), or control (Con, *n* = 6, Figure 1A).

The care, maintenance, and treatment of animals in these studies followed protocols approved by the Institutional Animal Care and Use Committee of the Korea Institute of Radiological and Medical Sciences (IACUC permit number: KIRAMS2019-0011, Approval date 2019-05-27). Experiments involving animals were performed according to the Guide for the Care and Use of Laboratory Animals published by the US National Institutes of Health. The animal housing chambers were automatically controlled at a temperature of 22 ± 3 °C and 55 ± 20% humidity under a 12 h light/dark cycle. A sterilized rodent diet and purified tap water were provided ad libitum.

### 4.2. Stress Exposure Protocol

**Figure 3:**
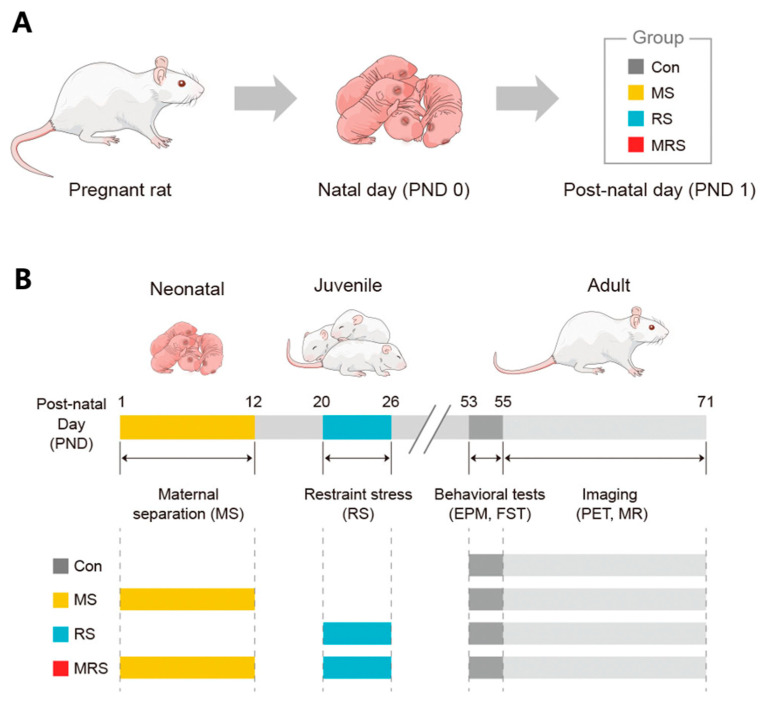
Classification of the animal groups (**A**) and schematic of the study protocol (**B**).

Stressor exposure for each group is shown in Figure 3. MS stress was performed during the neonatal period for the MS and complex trauma groups. At this stage, pups were removed from the home cage and placed inside incubators for 4 h (09:00–13:00) daily from PND 2 to PND 13. Rats were physically separated from each other. After a specific period of separation, the pups were individually returned to their home cages.

After a 1-week rest period (PND 13–19), the pups were weaned. The rats were then randomly re-housed under conventional housing conditions with two rats per cage. For restraint stress, the rats were returned to their home cages. The control group was not subjected to any stress during the experiments. All rats were housed under standard conditions on PND 51 (Figure 3B).

### 4.3. Behavioral Tests

#### 4.3.1. Elevated Plus Maze

Animals were tested for anxiety-like behavior in an elevated plus-maze according to previously described procedures [35]. The maze was made of acryls and consisted of two open arms (40 × 10 cm) and two closed arms with walls (40 × 10 × 30 cm) that extended from a common central platform (10 cm × 10 cm). The maze was placed at the center of the room and had similar levels of illumination (30 lux) in both the open and closed arms. Animals were individually placed on the central platform facing an open arm and allowed to freely explore the maze for 5 min. Behaviors were recorded with a video camera (Hubble 300, Screen For You Co, Kyoto, Korea) mounted vertically above the apparatus. Immediately after each session, the apparatus was cleaned with 80% ethanol. The time spent in the open and closed arms was analyzed using video-tracking software (SMART, Panlab Harvard apparatus, Barcelona, Spain).

#### 4.3.2. Forced Swim Test

The test was performed as previously described [36]. All experiments were performed in acrylic cylinders (diameter, 20 cm; height, 45 cm) filled with 35 cm of water. Rats were forced to swim for 15 min on the first day of the experiment (pretest day) to ensure that the animals adopted an immobile posture on the test day. Groups were reassessed 24 h later for 5 min, and the duration of immobility was recorded. Immobile behavior is believed to reflect a failure to persist in escape-directed behavior after stress and is positively correlated with depression [37]. Therefore, the duration of immobility is related to depression-like behavior. The water was changed between the experiments to ensure that rats were clearly visible. Water temperature was maintained at 24 ± 1 °C throughout the experiment. The immobility time was recorded using commercial software (SMART, Panlab Harvard Apparatus, Spain).

### 4.4. Molecular Imaging

#### 4.4.1. Preparation of Radiotracers

To evaluate alterations in brain circuits, including dopaminergic, glutamatergic, and serotonergic systems, three different types of radiotracers were employed. (S)-*N*-[(1 allyl-2-pyrrolidinyl)methyl]-5-(3[^18^F]fluoropropyl)-2,3-dimethoxybenzamide ([^18^F]fallypride) is a radiotracer for the estimation of the dopaminergic system (i.e., D2/3R). 3-[^18^F]fluoro-5-(2-pyridinylethynyl)benzonitrile, [^18^F]FPEB) is a radiotracer for evaluation of the glutamatergic system (i.e., mGluR5). *N*-{2-[4-(2-methoxyphenyl)piperazinyl]ethyl}-*N*-(2-pyridyl)-*N*-(*trans*-4-[^18^F]-fluoromethylcyclohexane)carbox amide, [^18^F]Mefway is a radiotracer for the serotonergic system (i.e., 5HT_1A_ receptor). All radiotracers were prepared according to previously described procedures [38,39,40]. The radiochemical purity of the radiotracers was >99%.

#### 4.4.2. PET/CT Scan

PET images of the rats were obtained using a small-animal PET scanner (nanoScan^®^, Mediso, Budapest, Hungary). The scanner had a peak absolute system sensitivity of >9% in the 250–750 keV energy window, an axial field of view of 28 cm, a transaxial field of view of 35–120 mm, and a transaxial resolution of 0.7 mm at 1 cm off-center. At the time of PET/CT scan, body weights of rats were as follows: MS = 388.7 ± 19.4 g, RS = 403.7 ± 39 g, MRS = 281 ± 48 g, Con = 402.9 ± 36.9 g.

Rats were anesthetized with 2.5% isoflurane in oxygen, and 13.7 ± 2.3 MBq of [18F]fallypride, [18F]FPEB and [18F]Mefway in 1 mL of saline was intravenously (i.v.) injected into the tail vein with a syringe pump (Pump Elite 11, 70-4500, Harvard) over 1 min. Before the injection of [18F]Mefway, aqueous fluconazole (60 mg/kg, OneFlu injection, JW Pharmaceutical, Korea) was pre-administered for 1 h to prevent the spillover from skull radioactivity.

Dynamic PET scanning was performed over 60 min with 24 frames (14 × 30 s, 3 × 60 s, 4 × 300 s, and 3 × 600 s). The image scans were acquired with an energy window of 400–600 keV. All images were reconstructed using the 3-dimensional ordered subset expectation maximization (3D-OSEM) algorithm with four iterations and six subsets. For attenuation correction and anatomical reference, micro-CT imaging was conducted immediately after PET using 50 kVp of X-ray voltage with 0.16 mAs.

#### 4.4.3. PET Image Analysis

For the analysis of rat brain data, a study-specific brain template was used. Each MR image was spatially normalized to the W. Schiffer T2-weighted rat brain MRI template using PMOD software (version 3.8, PMOD Technologies Ltd., Switzerland). Normalized brain MR images were summed, and a Gaussian filter (full width at half maximum (FWHM) = 1.0 mm) was applied to minimize noise-induced bias. A T2-weighted brain MR template was generated by masking the brain. After static PET images (4–26 frames) were obtained from the dynamic PET image, each PET image was co-registered to the raw MR image. The mean PET image was spatially normalized to the MR template. Finally, individual dynamic PET images for all groups were spatially normalized to the MR template, and brain-masking was applied.

The decay-corrected regional time–activity curves (TACs) were acquired from VOIs and normalized in units of standardized uptake value (SUV), which was calculated to normalize for the differences in the injected dose and body weight. The SUV obtained for each region of activity was multiplied by the body weight and divided by the injected dose. VOIs in PET images included the cortex, striatum, septum, and hippocampus; these VOIs were determined in the MR template. The striatum contained a high concentration of D2R and mGluR5. The cortex and hippocampus contained mGluR5 and 5-HT1A. The septum also contained 5-HT1A.

#### 4.4.4. MR Imaging

To define the anatomical VOIs, MR scans were obtained on a 31-cm horizontal-bore Agilent 9.4 T scanner (Agilent Technologies, Santa Clara, CA, USA) using a four-channel array rat head surface coil (Rapid Biomedical GmbH, Rimpar, Germany). The image parameters for the 3D turbo spin-echo (TSE) T2-weighted images were as follows: repetition time (TR) = 2500 ms; echo time (TE) = 7.45 ms; field of view (FOV) = 20 mm × 20 mm × 10 mm; matrix size = 128 × 128 × 64; voxel size = 156 μm × 156 μm × 156 μm; echo train length (ETL) = 43; and scan time = 1 h 54 min 50 s. During imaging, the respiratory rate of rats was monitored using an MR-compatible physiological monitoring and gating system (SA Instruments Inc., Stony Brook, NY, USA).

#### 4.4.5. Statistical Analysis

Quantitative results are expressed as means ± SEM. All statistical analyses were performed using GraphPad Prism software (version 8.0, GraphPad Software, Inc. San Diego, CA, USA). Differences between the groups in behavioral test and NeuroPET were tested using the Mann–Whitney nonparametric test, with *p* < 0.05 considered significantly different.

## Figures and Tables

**Table 1:** Body weights and corticosterone concentration at postnatal day (PND) 55.

Group	Body Weight (g)	Corticosterone (ng/mL)
Control	402.9 ± 36.9	196.5 ± 21.3
MS	388.7 ± 19.4 g	123.9 ± 16.8
RS	403.7 ± 39	111.7 ± 18.6
MRS	281 ± 48 **	138.8 ± 15.4

Data are presented as mean ± SEM (*n* = 5). Statistical significance was defined as a *p*-value less than 0.05 (** *p* < 0.01).

## Data Availability

The data presented in this study are available on request from the corresponding author.

## Supplementary Materials

The following are available online at https://www.mdpi.com/1422-0067/22/5/2522/s1.
