# Supplementary material for: Evaluation of the Effects of Developmental Trauma on Neurotransmitter Systems Using Functional Molecular Imaging
Source: Int J Mol Sci. 2021 Mar 3;22(5):2522. doi: 10.3390/ijms22052522 (PMC7959121; doi:10.3390/ijms22052522)
Supplement: Supplementary file 1 [file ijms-22-02522-s001.pdf]

**Supplementary Information**

# Evaluation of the effects of developmental trauma on neurotransmitter systems using functional molecular imaging

**Namhun Lee[1], Se Jong Oh[1], Jang Woo Park[2], Kyung Rok Nam[1], Kyung Jun Kang[1], Kyo Chul Lee[1], Yong Jin Lee[1], June-Seek Choi[3], Jeong-Ho Seok[4*] and Jae Yong Choi[1,5*]**

[1]Division of Applied RI, Korea Institute of Radiological and Medical Sciences, Seoul, Korea

[2]Radiological & Medico-Oncological Sciences, University of Science and Technology, Daejeon, Korea

[3]Medical Device-Bio Research institute, Korea Testing & Research Institute, Gyeonggi-do, Korea

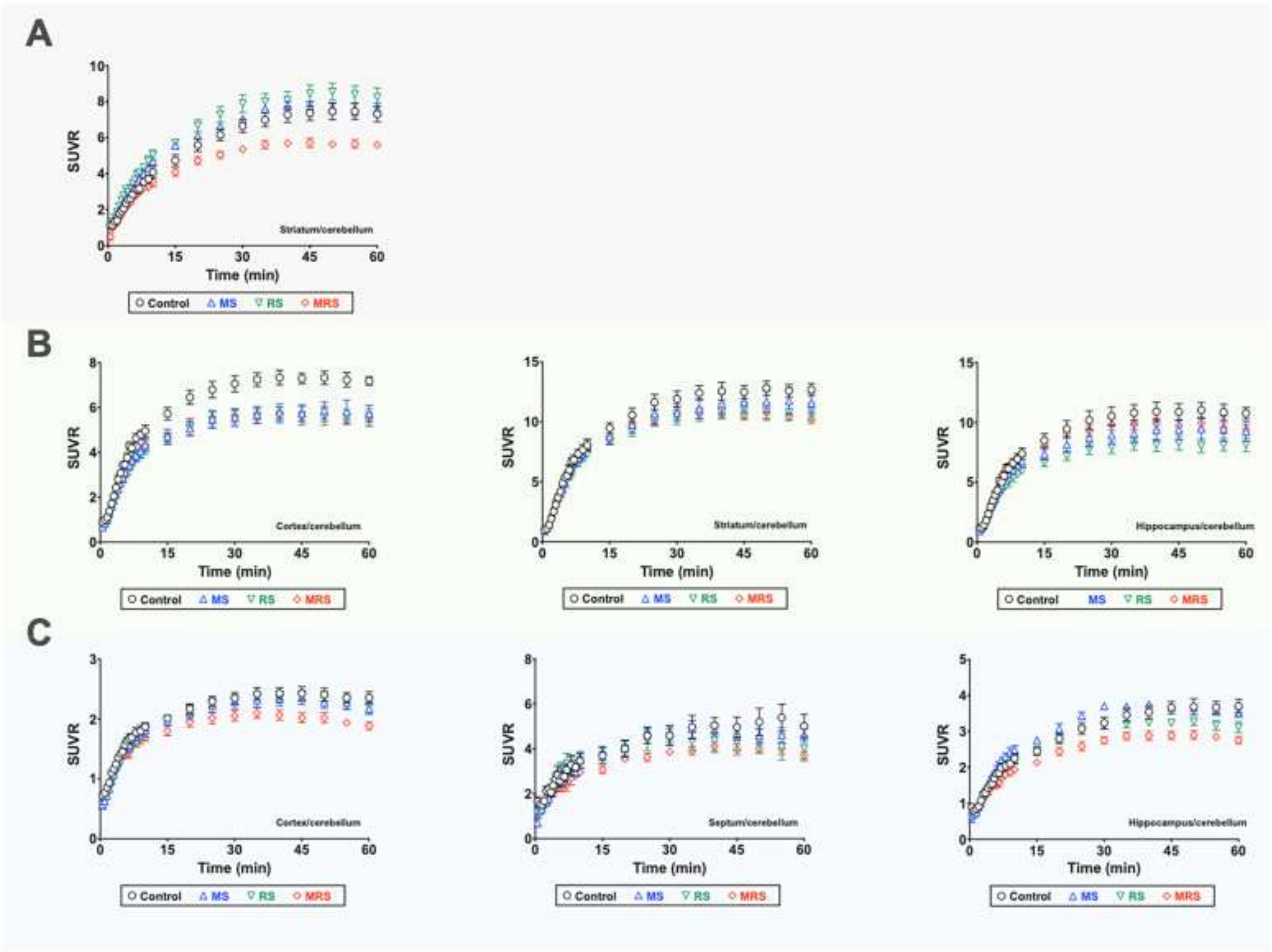


**Figure S1**. TACs for the cerebellum of the $^{18}$F-FP-CIT (A) and $^{18}$F-FPEB (B). Data were expressed as the mean ± the SD (n = 5).

**Table S1**. Comparison of the regional brain uptake value ratios in neuroPET

| Radiotracers | Group | Brain uptake value ratio (SUVR) | | | |
|---|---|---|---|---|---|
| | | Cortex/ cerebellum | Striatum/ cerebellum | Hippocampus/ cerebellum | Septum/ Cerebellum |
| [$^{18}$ F]fallypride for D2R | Con | – | 7.35 ± 0.33 | – | – |
| | MS | – | 7.56 ± 0.97 | – | – |
| | RS | – | 8.24 ± 0.38 | – | – |
| | MRS | – | 5.66 ± 0.27 | – | – |
| [$^{18}$ F]FPEB for mGluR5 | Con | 7.46 ± 0.31 | 13.10 ± 0.61 | 11.26 ± 0.69 | – |
| | MS | 5.94 ± 0.44* | 11.58 ± 0.90 | 9.46 ± 0.58 | – |
| | RS | 5.53 ± 0.22*** | 10.90 ± 0.44* | 8.43 ± 0.38** | – |
| | MRS | 5.47 ± 0.31*** | 10.00 ± 0.56** | 9.27 ± 0.26* | – |
| [$^{18}$ F]Mefway for 5-HT$_{1A}$ R | Con | 2.62 ± 0.08 | – | 5.09 ± 0.17 | 3.76 ± 0.33 |
| | MS | 2.37 ± 0.03* | – | 4.75 ± 0.16 | 3.33 ± 0.12 |
| | RS | 2.39 ± 0.05* | – | 4.44 ± 0.15* | 3.16 ± 0.31 |
| | MRS | 2.03 ± 0.09*** | – | 3.71 ± 0.19*** | 2.81 ± 0.17* |

Data are presented as mean ± SEM (n = 5). **p* < 0.05, ***p* < 0.01, ****p* < 0.01 compared to the control group.
